# Systemic Allergic Reactions to Subcutaneous Allergen Immunotherapy—A Single-Center Experience

**DOI:** 10.3390/life15101527

**Published:** 2025-09-28

**Authors:** Nataša Kusić, Aleksandra Plavšić, Vojislav Đurić, Jasna Bolpačić, Rajica Stošović, Milan Dimitrijević, Jelena Spirić-Milovančević, Irena Oštrić Pavlović, Antonije Veličković, Vesna Tomić-Spirić

**Affiliations:** 1 Clinic of Allergy and Immunology, University Clinical Center of Serbia, 11000 Belgrade, Serbia; aleksandra.plavsic@med.bg.ac.rs (A.P.); vojislav.djuric@med.bg.ac.rs (V.Đ.); jasna.bolpacic@med.bg.ac.rs (J.B.); rajica.stosovic@med.bg.ac.rs (R.S.); milandimitrijevic1411@gmail.com (M.D.); anerio89@gmail.com (I.O.P.); antonije.velickovic@gmail.com (A.V.); vesna.tomic-spiric@med.bg.ac.rs (V.T.-S.); 2 Faculty of Medicine, University of Belgrade, 11000 Belgrade, Serbia; 3 Clinic of Gastroenterology and Hepatology, University Clinical Center of Serbia, 11000 Belgrade, Serbia; spiricjelena77@gmail.com

**Keywords:** subcutaneous allergen immunotherapy, systemic allergic reactions

## Abstract

Background: Allergen immunotherapy (AIT) is generally considered a safe treatment modality, with systemic reactions (SRs) representing its most significant adverse events, despite their low incidence. This study aimed to evaluate the frequency and characteristics of SRs associated with subcutaneous allergen immunotherapy (SCIT) and to identify potential risk factors. Methods: We conducted a retrospective analysis of 47,982 SCIT injections administered to 317 patients over 468 SCIT courses between January 2019 and January 2024. The study population consisted of 26 patients diagnosed with allergic rhinitis sensitized to pollen and/or house dust mites (HDMs), as well as individuals with venom allergies who experienced SRs to SCIT during the study period. Data collected included demographic characteristics, presence of asthma, allergen sensitivities, immunoglobulin E (IgE)-related immunologic biomarkers, and adverse reactions. SRs were classified according to the World Allergy Organization (WAO) SCIT SR Grading System. Results: A total of 26 SCIT-related SRs were documented in 26 patients (57.7% female; mean age 37.3 ± 10.04 years), corresponding to an incidence rate of 0.05% per injection, and 8.2% per patient. Asthma was present in 42.3% of patients. Prior adverse reactions to SCIT were noted in eight patients (30.8%). SRs occurred during the build-up phase in 61.5% of cases, compared with the maintenance phase. In 46.2% of patients, a single allergen was administered, while 53.8% received multiple allergens. Based on the WAO grading system, 30.8% of SRs were classified as grade 1, 42.3% as grade 2, 15.4% as grade 3, and 11.5% as grade 4. No fatalities were reported. The majority of SRs were early onset (88.5%), and epinephrine was administered in 76.9% of the cases. A higher serum specific IgE to total IgE (sIgE/tIgE) ratio was significantly associated with more severe SRs. Conversely, a history of prior allergic reactions to SCIT appeared to correlate with milder SRs. Conclusions: Our findings confirm that SRs to SCIT are rare, and severe reactions are infrequent. A higher serum sIgE/tIgE ratio can be risk factor for severe SRs. Nonetheless, a thorough risk–benefit assessment is essential prior to initiating SCIT, particularly in patients with identified risk factors.

## 1. Introduction

Allergen immunotherapy (AIT) is the only treatment that targets the underlying causes of immunoglobulin E (IgE)-mediated allergic diseases, modifying their natural course and offering long-lasting benefits. It mediates its effects through modulation of the Th1/Th2 balance, induction of regulatory T cells (Treg), and shifts in allergen-specific IgE and IgG4 titers, which together contribute to sustained clinical efficacy, a reduced need for pharmacotherapy, and preventive effects [1,2]. Both subcutaneous (SCIT) and sublingual (SLIT) allergen immunotherapy are similarly effective and generally safe [3,4], although SLIT is associated with a significantly lower number of adverse reactions (ARs) [5,6,7,8]. Nevertheless, SCIT remains the most widely used and extensively studied AIT modality, providing higher adherence through structured clinical supervision [6] and remaining the preferred option in regions where SLIT is less accessible or not reimbursed.

The safety and efficacy of SCIT have been well established in the treatment of allergic respiratory diseases, as well as venom hypersensitivity [9,10,11,12,13], however the risk of ARs remain an important consideration. ARs are usually present as localized reactions (LRs) at the injection site. Nevertheless, SCIT also carries potential risks for systemic allergic reactions (SRs), which may range from mild cutaneous reactions to life-threatening or even fatal anaphylaxis [14]. SRs are rare when following a conventional protocol, with a prevalence of less than 1.0% of patients and 0.1% to 0.2% per injection visits [15,16,17]. It is estimated that life-threatening SRs occur once every 160,000 injection visits [18]. Fatal reactions to SCIT are extremely rare, with a rate of one fatal reaction per 7.2 million to 9.1 million injection visits [15,18].

Due to the potentially life-threatening nature of SRs, identifying risk factors for these reactions is crucial for ensuring patient safety during SCIT. Detailed surveys completed by clinicians reporting fatal reactions and near-fatal anaphylaxis have provided valuable insights into clinical presentations and contributing risk factors [19,20]. The most commonly reported risk factors for fatal reactions are uncontrolled asthma (62%), a history of prior SRs to SCIT (53%), administration during the peak allergy season (47%), the suboptimal management of anaphylaxis, including delayed epinephrine use (43%), and dosing errors (35%) [14].

The immunological characteristics of the patients who experienced SCIT-associated SRs remain unclear. Total serum IgE (tIgE) and specific IgE (sIgE) concentrations are primarily utilized to predict treatment efficacy in patients with allergic rhinitis and asthma [21,22,23]. Nevertheless, their potential utility in identifying patients at increased risk of SRs, particularly severe SRs, may also be considered.

In this study, we aimed to determine the frequency and characteristics of SRs to SCIT, to analyze the clinical and immunological characteristics of the patients who experienced SRs during SCIT treatment and to identify the potential patterns or predictors that could inform risk stratification and improve the safety of SCIT administration.

## 2. Materials and Methods

### 2.1. Patients

In this retrospective observational study, we analyzed 47,982 SCIT injections that were administered to 317 patients (188 males and 129 females) over 468 SCIT courses (90 patients were receiving more than one allergen extract) between January 2019 and January 2024 in the Diagnostic-polyclinic department of the Clinic of Allergy and Immunology, University Clinical Center of Serbia (UCCS). The study specifically focused on 26 patients who developed SRs during SCIT over the study period. These patients were diagnosed with allergic rhinitis, with or without asthma, sensitized to pollen or house dust mite (HDM) allergens, or had an insect venom allergy.

### 2.2. SCIT

Native allergen extracts used for SCIT were produced by the National Institute for Virology, Vaccines and Sera “Torlak” (Belgrade, Serbia) following the approved specifications and controlled protocols. Standardization involves quality control of the extract as the starting material for final immunotherapy preparation, in accordance with the requirements of the European Pharmacopoeia. This process comprises the evaluation of sterility, total protein content, and both protein and allergen profiles. Formulated in buffered saline and stabilized with aluminum phosphate, these non-modified allergen extracts have their potency expressed in protein nitrogen units (PNU)/mL and are prepared as named patient products (NPPs). For pollen and HDM extracts, three concentrations are produced (100, 1000, and 5000 PNU/mL for pollen; 10, 100, and 1000 PNU/mL for HDM), while venom extracts are formulated in four escalating strengths (1, 10, 100, and 1000 PNU/mL) (Supplementary Materials). The SCIT protocol consists of build-up phase, during which allergen doses are gradually increased on a weekly basis, until a maintenance dose is reached. This is followed by the maintenance phase, where the patients receive injections every 4 to 6 weeks over a period of 3 to 5 years. Following each SCIT injection, patients are observed for a minimum of 30 min to monitor for potential ARs.

The SCIT allergens extracts included in this study (pollens, HDMs, and insect venoms), represent the complete range of SCIT treatments provided at our Center during the study period. Prior to the initiation of SCIT, all the patients provided written informed consent.

### 2.3. Clinical and Imunological Parameters

All data were collected through patients’ medical records. We analyzed patient characteristics such as age, gender, sensitization, presence of asthma, usage of beta-blockers and angiotensin-converting enzyme (ACE) inhibitors, as well as history of anaphylaxis. We also recorded all ARs to SCIT that had occurred in these patients at any time prior to the analyzed SRs and defined them as previous ARs.

Immunological markers included tIgE and sIgE, expressed in international units per milliliter (IU/mL). Total IgE concentrations were measured using an enzyme-linked immunosorbent assay (ELISA). Specific IgE screening was initially performed using the RIDA^®^ qLine Allergy assay (R-Biopharm, Darmstadt, Germany), a line immunoassay based on nitrocellulose membranes that enables simultaneous semi-quantitative detection of IgE antibodies against a broad panel of allergens. Confirmatory and quantitative sIgE measurements reported in this study were obtained using the ImmunoCAP^®^ system (Thermo Fisher Scientific, Uppsala, Sweden), a fluorescent enzyme immunoassay (FEIA). All analyses were conducted at the Department for highly specialized in vitro diagnostics of immunological and allergic diseases, Clinic of Allergy and Immunology, UCCS. The concentrations of these immunological markers were used to confirm sensitization to relevant allergens but were not applied as selection criteria for initiating SCIT. All treatment decisions were based on clinical diagnosis and current guideline recommendations [24,25,26,27]. In polysensitized patients receiving SCIT with multiple allergens, several sIgE values were available. For the purposes of analysis, the highest sIgE value among the administered allergens was considered the dominant sIgE and was used in further statistical evaluation. The ratio of dominant sIgE to tIgE (sIgE/tIgE) was also calculated.

We recorded data regarding SCIT injections (allergen type, dose) and reaction details (clinical severity, onset timing, management of the reaction), as well. SRs were classified into five grades according to the World Allergy Organization (WAO) SCIT SR Grading System (Table 1) [17]. The classification was based on clinical manifestations observed following SCIT administrations and ranges from mild (grade 1), moderate (grade 2), severe (grade 3), and extremely severe or life-threatening reactions (grade 4), to fatal reactions (grade 5). Reactions that developed in the first 30 min were defined as early onset, and those that developed after 30 min were defined as delayed onset.

### 2.4. Statistical Analysis

Continuous variables with normal distribution were represented as mean ± standard deviation (SD), while non-normal distribution was represented by median and range (minimum and maximum values). Quantitative data among multiple groups were analyzed by ANOVA and Kruskal–Wallis tests. Categorical variables in terms of frequency and percentage were expressed and were compared using the Chi-square test. Ordinal logistic regression was performed to evaluate possible risk factors and results were reported as odds ratios (ORs) with 95% confidence intervals (CIs). Model fit was further assessed using Pearson and deviance goodness-of-fit tests, pseudo R^2^ indices, and the test of parallel lines. In addition, sensitivity analyses were conducted using binary logistic regressions with alternative dichotomizations of reaction severity (≥2 vs. ≤1; ≥3 vs. ≤2; grade 4 vs. ≤3) to explore the robustness of the findings. Statistical analyses were performed using SPSS version 23.0. A *p* value of <0.05 was considered statistically significant.

## 3. Results

### 3.1. Patient, SCIT and Systemic Reaction Characteristics

A total of 26 SCIT-associated SRs were observed in 26 patients (0.05% per injection, and 8.2% per patient). Statistical analyses were conducted at the reaction/patient level (n = 26). The mean age of the patients was 37.3 ± 10.04, with ages ranging from 19 to 67 years. More than half were female (57.7%, n = 15), while 42.3% were male (n = 11). Asthma was the most frequent comorbidity, in 42.3% of cases (n = 11). There was no significant difference in the number of patients with or without asthma (*p* = 0.433). Treatment with beta-blockers or ACE inhibitors was not recorded in any of the patients. None of the patients had a previous episode of anaphylaxis. Previous ARs to SCIT were observed in 8 of the 26 patients (30.8%) who experienced SRs during the five-year follow-up period. Among these, six patients (23.1%) had previously experienced LRs and two patients (7.7%) had mild SRs (which occurred prior to the study period). The remaining 18 patients (69.2%) had no documented SCIT-related ARs in their medical history. There was a statistically significant higher number of patients without a previous reaction, compared with those with a previous reaction (*p* = 0.050).

A single allergen content was administered in 46.2% of cases (n = 12), while multiple allergens were given in 53.8% (n = 14) of cases simultaneously. Two allergens were given to 26.9% (n = 7) of patients, three to 23.1% (n = 6), and four allergens were given to one patient (3.8%) simultaneously. Frequencies of administered allergens were as follows: weed pollen mix in 15 patients (57.7%), grass pollen mix in 14 patients (53.8%), HDMs in 12 patients (46.2%), tree pollen mix in 3 patients (11.5%), and insect venoms in 2 patients (7.7%). Sixteen SRs occurred during the build-up phase of SCIT (61.5%), while ten were observed during the maintenance phase (38.5%) (Table 2).

**Figure 1:**
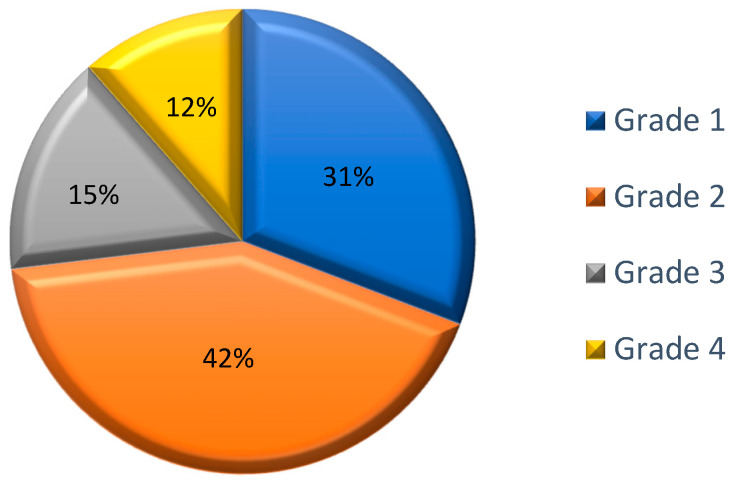
Classification of the systemic reactions according to the WAO SCIT SR Grading System; Data are expressed as percentages of the total number of observed SRs (n = 26).

The majority of SRs involved cutaneous symptoms (80.8%, n = 21), with respiratory symptoms also frequently reported (57.7%, n = 15). Most of the SRs were early onset (88.5%, n = 23). Epinephrine was administered in the majority of cases (76.9%, n = 20). According to the WAO SCIT SR Grading System, 30.8% (n = 8) of the SRs were classified as grade 1; 42.3% (n = 11) as grade 2; 15.4% (n = 4) as grade 3; and 11.5% (n = 3) as grade 4. The incidence of life-threatening SRs (grade 4) was 0.006% per injection. There was no fatal outcome from any of the SRs. (Table 3, Figure 1).

### 3.2. Factors Associated with Reaction Severity

There was no statistically significant difference in the mean age of patients across different severity groups of SRs (one-way ANOVA).

We found no statistically significant association between the number of simultaneously administered allergens (one vs. multiple) and reaction severity (Chi-square test, *p* = 0.619). In the single-allergen group, grade 2 reactions were most common (50%), while in the multiple-allergen group, grades 1 and 2 were equally frequent (35.7% each). Grade 4 reactions were rare in both groups (16.7% vs. 7.1%).

**Figure 2:**
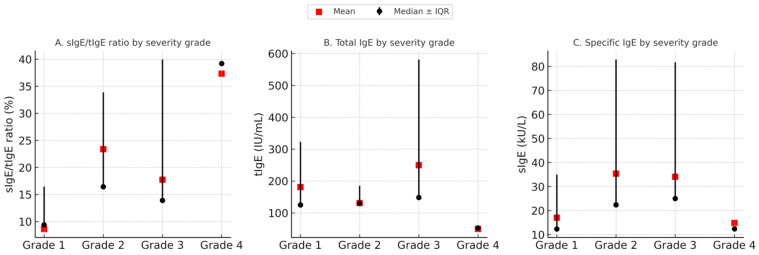
Comparison of immunological parameters across systemic reaction severity grades. Median values of tIgE, sIgE, and the sIgE/tIgE ratio are shown for each severity grade (●), with error bars representing interquartile ranges (IQRs). Mean values are indicated by 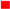. A statistically significant difference was observed in the sIgE/tIgE ratio between mild and extremely severe reactions (*p* = 0.014), while no significant differences were found for tIgE and sIgE alone.

The Kruskal–Wallis test was performed to estimate if there was a difference in the mean values of tIgE, sIgE, or their ratio (sIgE/tIgE) in regard to severity of reaction. Results showed statistically significant differences in the ratio values between the groups with different severities of SRs. With further post hoc analysis (Bonferroni correction), we observed statistically significant differences in mean values of sIgE/tIgE when comparing groups with mild (grade 1) and extremely severe reactions (grade 4) with the highest values of ratio observed in patients with grade 4 reactions (9.4 (3.29–13.33) vs. 39.2 (16.3–56.5), *p* = 0.014) (Figure 2).

### 3.3. Comparation of Immunological Parameters According to Clinical Characteristics

There was no statistically significant difference in mean values of tIgE, sIgE, and sIgE/tIgE based on the presence of asthma, gender, the phase of SCIT, and the number of administered allergens.

Statistically significant differences in the mean values of these parameters were, however, observed with regard to the presence of previous reactions—patients with previous reactions had a higher tIgE (157 (120–410) vs. 99.5 (20–620), *p* = 0.02); sIgE (40.5 (15.3–102) vs. 10.35 (3.5–78), *p* = 0.009) and sIgE/tIgE ratio (15.79 (11.7–64.96) vs. 9.56 (3.29–56.5), *p* = 0.04).

### 3.4. Predictors of Severe Systemic Reactions

Ordinal logistic regression was performed to analyze the association between various clinical and immunological factors and the severity of allergic reactions.

Among the included predictors, sIgE/tIgE was found to be a significant positive predictor of more severe allergic reactions (OR = 1.19, 95% CI: 1.00–1.41, *p* = 0.049). This indicates that higher sIgE/tIgE ratios are associated with increased odds of experiencing more severe reactions.

Previous allergic reactions related to SCIT showed a borderline protective effect (OR = 0.10, 95% CI: 0.01–1.05, *p* = 0.055), suggesting a potential trend toward milder reactions in patients with a history of previous reactions.

Other variables, including tIgE (OR = 1.01, 95% CI: 0.99–1.02, *p* = 0.191), sIgE (OR = 0.93, 95% CI: 0.84–1.03, *p* = 0.190), and the presence of asthma (OR = 0.31, 95% CI: 0.05–1.89, *p* = 0.203) were not statistically significant predictors in this model.

Model fit was evaluated using multiple indices. The Pearson goodness-of-fit test was non-significant (χ^2^ = 63.396, df = 66, *p* = 0.568), as was the deviance test (χ^2^ = 50.361, df = 66, *p* = 0.923), indicating adequate overall fit of the model. Pseudo R^2^ values suggested that the model explained a moderate proportion of the variance in reaction severity (Cox & Snell = 0.384, Nagelkerke = 0.417, and McFadden = 0.191). The test of parallel lines, however, was significant (χ^2^ = 51.747, df = 6, *p* < 0.001), indicating that proportional-odds assumption was not met. This result suggests that the estimated odds ratios may not be strictly constant across all thresholds of the outcome. Given the small sample size and limited number of grade 4 events, alternative models such as generalized ordered logit were unstable. We therefore interpreted the proportional-odds estimates with caution and considered them as hypothesis-generating findings that warrant confirmation in larger samples.

As a sensitivity analysis, we repeated the regression using binary logistic models with alternative dichotomizations of reaction severity (≥2 vs. ≤1; ≥3 vs. ≤2; grade 4 vs. ≤3). Across all models, the direction of the sIgE/tIgE effect remained consistent with the ordinal regression, indicating that higher ratios were associated with increased odds of more severe reactions (OR ≈ 2.13, 95% CI: 0.83–5.49, *p* = 0.118). Although the association did not reach statistical significance in these smaller models, the robustness of the effect direction supports the potential relevance of this parameter.

## 4. Discussion

Our study demonstrates that SCIT is generally safe, with a low incidence of SRs (0.05%) per injection. These results are in agreement with previously published data, although the incidence observed in our study is slightly lower compared with certain studies [7,8,15,16,17,28,29,30]. Severe, potentially life-threatening SRs remain exceptionally rare. Additionally, no fatality has been observed in our study.

According to the existing literature, systemic and other ARs to SCIT occur more frequently in female patients [31,32,33]. In our study, 15 out of 26 patients (57.7%) who experienced SCIT-associated SRs were female. However, other studies have reported no significant sex-based differences in the incidence of adverse reactions [34,35]. These conflicting findings underscore the need for further research, particularly to explore the potential role of hormonal and immunological factors in modulating the risk of SRs to AIT.

The presence of asthma is recognized as a significant risk factor for the development of SRs during AIT [20,36]. Data from the North American survey indicated that asthma was present in approximately two-thirds of patients who experienced grade 3 or 4 SRs [15]. In our study asthma was the most frequently observed comorbidity, identified in 42.3% of patients. However, no statistically significant difference was found in the incidence of SRs between patients with and without asthma (*p* = 0.433). Furthermore, asthma was not identified as a statistically significant predictor of severe SRs (OR = 0.31, 95% CI: 0.05–1.89, *p* = 0.203). This may be attributable to the exclusion of patients with uncontrolled asthma from SCIT initiation, as well as the confirmation of asthma control prior to each SCIT administration, which was assessed using anamnestic data (including symptom frequency, use of reliever therapy, and recent exacerbation history), physical examination (including auscultation), and pulmonary function testing when indicated. These findings align with previous studies suggesting that well-controlled asthma does not significantly increase the risk of SRs during SCIT [31].

Notably, none of our patients were undergoing treatment with beta-blockers or ACE inhibitors at the time of their reactions. While these medications have been considered as potential risk factors for severe anaphylactic reactions, recent evidence indicates that their use does not necessarily increase the frequency or severity of SRs during AIT [37].

In our study, 30.8% of patients who experienced SCIT-related SRs had a history of prior ARs to SCIT, predominantly LRs, while the majority (69.2%) did not experience any SCIT-related ARs before. The statistically significant higher number of patients without previous reactions (*p* = 0.050) suggests that prior ARs may not be predictive of subsequent SRs. These findings are consistent with the study by Yang et al. who reported that the incidence of SRs did not significantly increase in patients with a history of LRs, although the SR rate was higher when a LR or a large LR (LLR) preceded the injection. They concluded that individual LRs did not appear to be predictive of subsequent SRs, however some individuals with a higher frequency of LRs might be at greater risk of SRs [38]. Although LLRs were not found to predict subsequent SRs in a single retrospective practice study, 42% of patients who experienced LLRs also experienced SRs versus 11% without LLRs [39]. This finding was confirmed in a similar study by Roy et al. [40]. Nevertheless, a study that evaluated the impact of adjusting allergen doses for patients with and those without LLRs, found no differences in the rates of subsequent SRs [40]. While some studies suggest that previous LRs may not be reliable predictors of SRs, others indicate a potential association, particularly with LLRs [41]. These findings emphasize the complexity of predicting SRs based solely on prior ARs.

Interestingly, a borderline protective effect was observed in our patients with a history of previous SCIT-related reactions, suggesting a potential trend toward milder reactions in patients with a history of previous reactions. It may indicate that previous experience with ARs prompts greater caution in treatment (e.g., dose adjustment, closer monitoring), thereby reducing the risk of more severe subsequent reactions. This unexpected trend highlights the importance of individualized treatment planning in SCIT.

It has been well documented that venom immunotherapy (VIT) carries a higher risk of severe systemic reactions, especially in patients with clonal mast cell disorders such as systemic mastocytosis or monoclonal mast cell activation syndrome [42,43]. In our study, only a small proportion of SRs (7.7%) were triggered by venom extracts and none of the patients had a known mast cell disorder. Given the limited number of such cases in our cohort, firm conclusions cannot be drawn; however, venom allergy and mast cell-related conditions are well recognized as clinically important risk factors that require careful pre-treatment evaluation and close monitoring during SCIT.

The baseline serum tryptase has been identified as an important biomarker of risk for severe SRs, particularly in patients with venom allergies and in those with clonal mast cell disorders, where elevated levels are associated with a higher likelihood of such reactions [44]. While tryptase was not systematically measured in our study, its role should be emphasized in future research and clinical practice.

Although the administration of multiple allergens in SCIT is a common practice among polysensitized patients, it warrants careful evaluation due to the potential increased risk of SCIT-related SRs. Nacaroğlu et al. reported a significantly higher risk of ARs in patients receiving SCIT with multiple allergens concurrently [45]. In contrast, other studies have demonstrated a higher incidence of ARs among mono-sensitized patients [7]. In our study, 53.8% of patients received multiple allergens simultaneously, while a single allergen content was administered in 46.2% cases. We found no statistically significant association between the number of simultaneously administered allergens and reaction severity.

Previous studies have demonstrated that ARs to AIT tend to occur more frequently and with greater intensity during the build-up phase, compared with the maintenance phase [7,31,32,34,36,45,46]. Asllani et al. have found that reactions become quite rare after 6 weeks of maintenance, suggesting that a close observation period should be planned with that time frame in mind [7]. The majority of SRs (61.5%) observed in our study occurred during the build-up phase of SCIT, which is consistent with the existing literature data.

In a multicenter study by Calderon et al. in Germany, France, and Spain, 41.3% of SRs occurred in the first 30 min, 8.3% between 30 and 60 min, 14.7% between 60 and 120 min, and 35.8% improved after 120 min [34]. While most of the SRs in our study occurred within 30 minutes after SCIT administration (88.5%), there were still some SRs that developed later, including one after 90 minutes. Extending the observation period after injection and/or prescribing epinephrine autoinjectors in the event of delayed-onset anaphylaxis may be considered in the future.

The high rate of early-onset reactions emphasizes the necessity to promptly identify and treat potential severe SRs. Epinephrine was administered in 76.9% of our cases, reflecting the adherence to recommended guidelines for the management of SRs [47,48]. Importantly, all reactions in our study were managed effectively, with no fatalities reported.

Although IgE-related biomarkers are used to assess sensitization and monitor AIT efficacy, their role in predicting SR severity is less clear. Previous studies have associated high sIgE concentrations and skin test reactivity with increased SR risk [49,50,51,52,53,54,55]. Our analysis showed that patients with previous SCIT-related ARs had higher mean tIgE, sIgE, and sIgE/tIgE ratios compared with those without previous reactions. Notably, the sIgE/tIgE ratio differed significantly between mild and severe SR groups, with a higher ratio linked to increased odds of severe reactions (OR = 1.19, 95% CI: 1.00–1.41, *p* = 0.049). In contrast, tIgE and sIgE alone were not significantly associated with SR severity, consistent with the literature supporting the sIgE/tIgE ratio as a more reliable predictor of clinical response to AIT [21,22,23]. This distinction suggests that tIgE and sIgE may reflect an underlying atopic profile or prior immune activation, while the sIgE/tIgE ratio may serve as a more informative indicator of current immunological reactivity and the risk of more severe systemic responses. Notably, in our study, this ratio was not associated with baseline clinical characteristics, suggesting that it may provide additional immunological insight beyond routine clinical assessment. Consequently, our findings highlight the potential utility of the sIgE/tIgE ratio as a biomarker for risk stratification during SCIT. Incorporating this ratio into pre-treatment assessments may optimize patient selection and improve safety.

The limitations of our study include a single-center experience, a small sample size with few severe reactions reducing statistical power, a retrospective design, reliance on previously recorded data, and the expression of allergen extract potencies in PNU/mL. Nevertheless, the study still contributes valuable insights into the current understanding of SCIT-related SRs.

## 5. Conclusions

Our findings confirm that SCIT-related SRs are rare, with a low incidence of life-threatening SRs. An individualized approach and mitigation of known risk factors may further reduce the occurrence of SRs, while the sIgE/tIgE ratio may serve as a useful biomarker for identifying patients at higher risk of severe SRs and for guiding closer monitoring and dose adjustment.

This long-term real-world study reinforces the safety of SCIT and supports its safe application under appropriate clinical supervision.

## Figures and Tables

**Table 1:** World Allergy Organization grading system for systemic reactions with subcutaneous immunotherapy *.

Grade	
1	Symptom(s) and/or sign(s) of one organ system present: generalized pruritus, urticaria with and/or without angioedema (not laryngeal, tongue, or uvular); ORupper respiratory symptoms (e.g., rhinitis or itchy throat or cough originate in the upper airway); OR conjunctival symptoms; OR nausea
2	Symptom(s) and/or sign(s) of more than one organ system present; OR asthma that responds to an inhaled bronchodilator; OR gastrointestinal symptoms, including abdominal cramps, vomiting, or diarrhea; OR uterine cramps
3	Lower respiratory—severe asthma that does not respond to a bronchodilator; OR upper respiratory-laryngeal, uvular, or tongue edema, with or without stridor
4	Respiratory failure OR hypotension with or without loss of consciousness
5	Fatal anaphylaxis

* Adapted from Ref. [17].

**Table 2:** Previous reactions and SCIT characteristics (n = 26).

**Previous reactions**	**n (%)**
Previous adverse reaction to SCIT	8 (30.8)
- Local reactions	6 (23.1)
- Systemic reactions	2 (7.7)
Previous anaphylaxis	0 (0)
**SCIT characteristics**	**n (%)**
Single-allergen treatment	12 (46.2)
Multiple-allergen treatment	14 (53.8)
- two allergens	7 (26.9)
- three allergens	6 (23.1)
- four allergens	1 (3.8)
Type of administered allergen extract	
- weed pollen mix	15 (57.7)
- grass pollen mix	14 (53.8)
- tree pollen mix	3 (11.5)
- HDMs	12 (46.2)
- insect venoms	2 (7.7)
Build-up phase	16 (61.5)
Maintenance phase	10 (38.5)

Abbreviations: HDMs—House dust mites; SCIT—subcutaneous allergen immunotherapy.

**Table 3:** Systemic reaction characteristics (n = 26).

SR Characteristics	n (%)
Time of onset	
- Early onset (≤30 min)	23 (88.5)
- Delayed onset (>30 min)	3 (11.5)
Most frequently reported symptoms	
- Cutaneous	21 (80.8)
Respiratory	15 (57.7)
Epinephrine administration	
- Yes	20 (76.9)
- No	6 (23.1)
Severity (WAO SR Grading System)	
- Grade 1	8 (30.8)
- Grade 2	11 (42.3)
- Grade 3	4 (15.4)
- Grade 4 (life-threatening)	3 (11.5)
- Grade 5 (fatal outcome)	0 (0)

Abbreviations: SR—systemic reaction; WAO—World Allergy Organisation.

## Data Availability

The original contributions presented in the study are included in the article; further inquiries can be directed to the corresponding author.

## Supplementary Materials

The following supporting information can be downloaded at: https://www.mdpi.com/article/10.3390/life15101527/s1, SCIT build-up protocol.
