# Supplementary material for: Systemic Allergic Reactions to Subcutaneous Allergen Immunotherapy—A Single-Center Experience
Source: Life (Basel). 2025 Sep 28;15(10):1527. doi: 10.3390/life15101527 (PMC12565005; doi:10.3390/life15101527)
Supplement: Supplementary file 1 [file life-15-01527-s001.zip › life-3854033-supplementary.pdf]

SCIT build-up protocol

a) Pollen allergen extracts

100 PNU/ml	1000 PNU/ml	5000 PNU/ml
0.1 mL	0.1 mL	0.1 mL
0.2 mL	0.2 mL	0.2 mL
0.3 mL	0.3 mL	0.3 mL
0.4 mL	0.4 mL	0.4 mL*

* usual maintenance dose is 0.4ml of 5000PNU/ml concentration

b)HDM allergen extracts

10 PNU/ml	100 PNU/ml	1000 PNU/ml
0.1 mL	0.1 mL	0.1 mL
0.2 mL	0.2 mL	0.2 mL
0.3 mL	0.3 mL	0.3 mL
0.4 mL	0.4 mL	0.4 mL*

* usual maintenance dose is 0.4ml of 1000PNU/ml concentration

c) Hymenoptera venom extracts

1 PNU/ml	10 PNU/ml	100 PNU/ml	1000 PNU/ml
0.1 mL	0.1 mL	0.1 mL	0.1 mL
0.2 mL	0.2 mL	0.2 mL	0.2 mL
0.3 mL	0.3 mL	0.3 mL	0.3 mL
0.4 mL	0.4 mL	0.4 mL	0.4 mL*

*** usual maintenance dose is 0.4ml of 1000PNU/ml concentration**
